# Sudden death in a patient with long QT syndrome presenting with an epileptic phenotype

**DOI:** 10.1111/anec.12753

**Published:** 2020-03-21

**Authors:** Esseim Sharma, Stephen Gannon, Brian McCauley, Antony F. Chu

**Affiliations:** ^1^ Department of Electrophysiology Rhode Island Hospital Brown University Providence RI USA; ^2^ Department of Cardiology Brigham and Women’s Hospital Harvard University Boston MA USA; ^3^ Department of Cardiology University of Pennsylvania Philadelphia PA USA

**Keywords:** cardiac arrest, clinical ventricular, electrophysiology, fibrillation, long QT syndrome, sudden death, tachycardia

## Abstract

Patients with epilepsy suffer from a higher mortality rate than the general population, a portion of which is not due to epilepsy itself or comorbid conditions. Sudden unexpected death in epilepsy (SUDEP) is a common but poorly understood cause of death in patients with intractable epilepsy and often afflicts younger patients. The pathophysiology of SUDEP is poorly defined but does not appear to be related to prolonged seizure activity or resultant injury. Interestingly, a subset of patients with confirmed long QT syndrome (LQTS) present with a seizure phenotype and may have concurrent epilepsy. In this case, we present a patient who initially presented with a seizure phenotype. Further workup captured PMVT on an outpatient event monitor, and the patient was subsequently diagnosed with LQTS1. A substantial number of patients with LQTS initially present with a seizure phenotype. These patients may represent a subset of SUDEP cases resulting from ventricular arrhythmias. Appropriate suspicion for ventricular arrhythmias is necessary for proper arrhythmia evaluation and management in patients presenting with epilepsy.

## 1 INTRODUCTION

Patients with epilepsy suffer from a higher mortality rate than the general population. A portion of the mortality is attributable to prolonged seizures with or without physical injury and some due to other underlying medical conditions. (Nashef & Sander, 1996) There is however a portion of deaths which occur suddenly, without warning and appear unrelated to epilepsy itself or other comorbidities. Sudden unexpected death in epilepsy (SUDEP) is a common cause of death in patients with intractable epilepsy, accounting for 10%–50% of deaths. (Shorvon & Tomson, 2011; Surges, Thijs, Tan, & Sander, 2009; Tomson, Nashef, & Ryvlin, 2008) It was first defined by Nashef in 1997 as “Sudden, unexpected, witnessed or unwitnessed, nontraumatic, and nondrowning death in patients with epilepsy, with or without evidence of a seizure and excluding documented status epilepticus, in which postmortem examination does not reveal a toxicologic or anatomic cause for death.” (Nashef, 1997).

The devastating nature of the disease and young patient population, typically affecting those from 20 to 40 years of age, has resulted in significant interest in identifying risk factors and disease mechanisms. Identified risk factors include high frequency of generalized tonic‐clonic seizures, multiple antiepileptic drugs, male sex, duration of epilepsy, and associated learning disability. (Jones & Thomas, 2017) The pathophysiology of SUDEP is poorly defined but does not appear to be related to prolonged seizure activity or resultant injury. Proposed mechanisms include central induced respiratory dysfunction, postictal bradycardia and/or asystole, postictal generalized EEG suppression (PGES), and channelopathy mediated ventricular arrhythmias. The current data available suggest that ventricular arrhythmias account for a minority of deaths. (Ryvlin et al., 2013).

Interestingly, a subset of patients with confirmed long QT syndrome (LQTS) present with a seizure phenotype and may have concurrent epilepsy. Seizures are more common in patients with LQTS 2 than the other known genotypes. (Anderson, Bos, Cascino, & Ackerman, 2014) The role of ventricular arrhythmias in episodes of SUDEP may be underestimated in patients with concurrent LQTS. Here, we present a case of cardiac arrest in a patient with epilepsy who was found to have concurrent LQTS.

## 2 CASE PRESENTATION

A 42‐year‐old woman with a past medical history of migraines and seizures refractory to antiseizure medications was referred to cardiology for management of newly diagnosed nonischemic cardiomyopathy. Her family history was notable for premature coronary artery disease in her father, but there was no family history of sudden cardiac death or epilepsy. She had initially been diagnosed with epilepsy a year prior after having a witnessed event at home that involved loss of consciousness, upward eye‐rolling, and bilateral upper extremity convulsions lasting about 20 s, along with a prolonged postictal phase and aphasia. Her medications at the time included topiramate, amitriptyline, and sumatriptan. Workup included an EEG that showed nonspecific, mild‐to‐moderate left‐sided slowing, and a brain MRI that showed no structural abnormalities. Her seizures were thought to be related to a recent concussion she had suffered in the setting of a motor vehicle accident. She was seen by a neurologist, and her topiramate dosing, which she was previously taking for migraines, was intensified from 200 to 400 mg daily to also help manage her epilepsy.

**Figure 1:**
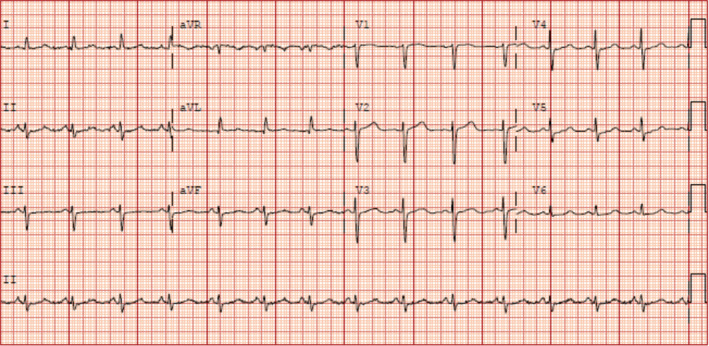
Baseline ECG showing normal sinus rhythm and a prolonged QTc interval of 510 milliseconds, taken during patient's second hospital admission

Two months later, the patient was hospitalized after another motor vehicle accident that was caused by a presumed seizure event that caused loss of consciousness. A high‐sensitivity troponin I was also checked on presentation, which peaked at 3.327 ng/ml. Baseline ECGs revealed normal sinus rhythm, rare premature ventricular complexes, and a variable QTc interval of 460–510 ms, one of which is shown in Figure 1. Workup during the hospitalization included a repeat EEG, which showed focal, left‐sided slowing but no epileptiform activity, and a transthoracic echocardiogram, which revealed moderately reduced left ventricular function with an ejection fraction (EF) of 35% with predominantly inferobasilar hypokinesis. A cardiac catheterization was subsequently performed and showed no significant coronary artery disease, and a presumptive diagnosis of stress‐induced nonischemic cardiomyopathy (NICM) was made. She was discharged on guideline‐directed medical therapy (GDMT) for heart failure and topiramate and levetiracetam for seizures.

**Figure 2:**
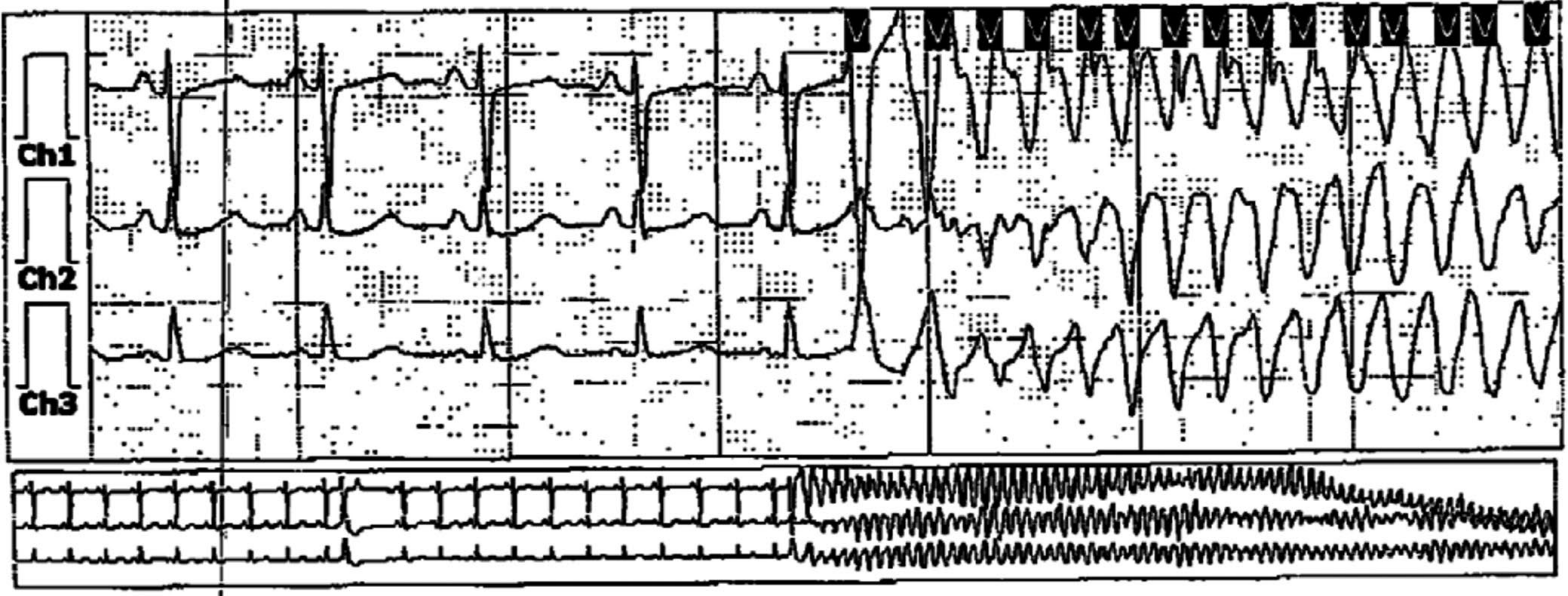
Electrographic recording from the patient's event recorder demonstrating an episode of polymorphic ventricular tachycardia

The patient was subsequently seen in cardiology clinic in follow‐up. A cardiac MRI was performed after three months of GDMT for heart failure and demonstrated an improved LVEF of 45%, apical hypokinesis, and no late gadolinium enhancement. She continued to complain of intermittent palpitations, so an outpatient event monitor was placed. During sleep, the patient had a witnessed grand mal seizure lasting for six minutes, during which she was unresponsive. She received bystander CPR and awoke spontaneously after a few minutes. The patient was wearing the event monitor at the time of her seizure revealing a six‐minute episode of polymorphic ventricular tachycardia (PMVT) initiated by a premature ventricular contraction leading to an R‐on‐T event (Figure 2), which spontaneously resolved. Medications at the time included metoprolol, levetiracetam, topiramate, and eslicarbazepine.

She was brought to the hospital for further workup of her PMVT. ECG on presentation to the hospital again showed a prolonged QT interval, this time to 508 milliseconds, and her laboratory workup was notable for a potassium level of 2.8 and a magnesium level of 1.6 millimoles per liter. Despite correction of her electrolyte abnormalities and cessation of QT‐prolonging medications, her QTc remained > 500 milliseconds, so genetic testing was sent for LQTS. An implantable cardiac defibrillator (ICD) was subsequently placed for secondary prevention of sudden cardiac death. Genetic testing eventually returned positive for LQTS1, and the patient's beta‐blocker was changed from metoprolol to the nonselective nadolol.

## 3 DISCUSSION

The misdiagnosis of epilepsy in patients with LQTS has been described in multiple case reports. (Ballardie, Murphy, & Davis, 1983; O'Callaghan & Trump, 1993; Schwartz, Moss, Vincent, & Crampton, 1993) Because of the relative infrequency of the LQTS, data are sparse in this population. Patients with LQTS often present with syncopal events with stereotypical movements that can mimic seizures; in a retrospective study by Johnson et al., 29% of 343 patients with LQTS presented with a seizure phenotype. (Johnson et al., 2009) Misdiagnosis can lead to prolonged, unnecessary workup, mismanagement, and even death. However, in one observational study of 40 patients, 34% of patients with long QT syndrome had baseline EEG abnormalities. (González et al., 2018) These baseline EEG abnormalities further cloud accurate diagnosis in patients with LQTS and make a delay in appropriate referral and treatment more likely.

Further complicating the diagnostic picture is the observation that LQTS can also coexist with true epilepsy in the same patient. In a retrospective study of 610 patients with LQTS, 11% of patients had seizures or seizure‐like episodes and 1.6% had been formally diagnosed with epilepsy by an epileptologist based on clinical and EEG findings. (Anderson et al., 2014) In a separate postmortem study of 68 SUDEP cases, mutations in genes associated with LQTS were found in 13% of patients. (Tu, Bagnall, Duflou, & Semsarian, 2011) Some authors have proposed a link between true epilepsy and cardiac arrhythmias in LQTS due to the underlying channelopathies affecting both the cardiac and neuronal tissues. (Bagnall, Crompton, & Semsarian, 2017).

Clearly, the relationship between epilepsy and LQTS is complex, and more research is necessary at the genetic and molecular level to elucidate the relationship between these two syndromes. Clinically, our case highlights the difficulty in distinguishing between the two entities. Our patient likely was misdiagnosed with epilepsy on the basis of clinical history and nonspecific EEG findings. A high suspicion for arrhythmia led to the capture of polymorphic VT on event monitor, confirmation of LQTS, and ICD placement. Over the next three years, the patient's antiepileptic drugs were weaned off, and she has had no further seizure events. Additionally, she has not had any further ventricular arrhythmias requiring ICD therapy either, likely due to the beneficial effect of nonselective beta‐blockers in LQTS1, as well as the cessation of QT‐prolonging medications and electrolyte repletion. (Chockalingam, 2012; Zipes, 2006).

## 4 CONCLUSIONS

Patients with epilepsy have a higher mortality rate than the general population, a portion of which is not due to epilepsy itself or comorbid conditions. Prior observational studies have shed some light on potential sudden death mechanisms; however, these occurred in highly selected populations. A portion of LQTS patients presents with a seizure phenotype, raising the possibility of misdiagnosis or concurrent epilepsy. These patients may represent a subset of SUDEP cases resulting from ventricular arrhythmias. Appropriate arrhythmia evaluation and management is critical to patients presenting with epilepsy. Testing for LQTS should be strongly considered in patients with epilepsy found to have documented ventricular arrhythmias and a prolonged QTc.

## CONFLICT OF INTEREST

The authors declare that there is no conflict of interest regarding the publication of this article.

## AUTHOR CONTRIBUTIONS

All authors have made substantial contributions to the conception and design of this case report. They have been involved in the drafting of the manuscript and revising it critically. They have given their final approval of the version to be published. Finally, they have agreed to be accountable for all aspects of the work in ensuring that questions related to the accuracy or integrity of any part of the work are appropriately investigated and resolved.

## ETHICS

Additionally, the authors have obtained the patient's free informed consent for the publication of this case report per the journal's ethical guidelines.
